# The risk profile of electronic nicotine delivery systems, compared to traditional cigarettes, on oral disease: a review

**DOI:** 10.3389/fpubh.2023.1146949

**Published:** 2023-05-15

**Authors:** Qing Zhang, Cai Wen

**Affiliations:** ^1^Department of Nosocomial Infection Control, The Affiliated Hospital of Southwest Medical University, Luzhou, Sichuan, China; ^2^Department of Oral Implantology, The Affiliated Stomatological Hospital of Southwest Medical University, Luzhou, Sichuan, China; ^3^Department of VIP Dental Service, The Affiliated Stomatological Hospital of Southwest Medical University, Luzhou, Sichuan, China; ^4^Luzhou Key Laboratory of Oral and Maxillofacial Reconstruction and Regeneration, The Affiliated Stomatological Hospital of Southwest Medical University, Luzhou, Sichuan, China; ^5^Institute of Stomatology, Southwest Medical University, Luzhou, Sichuan, China

**Keywords:** electronic nicotine delivery systems, tobacco, oral disease, e-cigarette, stomatology

## Abstract

The use of electronic nicotine delivery systems (ENDS) has exploded, especially among teenagers and new smokers, amid widespread awareness of the dangers of traditional tobacco and restrictions on smoking. However, the risk effects of ENDS on physical health, especially oral health, are still ambiguous. The purpose of this study was to review the available evidence on risks of ENDS on oral health, and compares the differences between ENDS and traditional cigarettes. For heavy smokers, transferring the addiction of tobacco to ENDS can be less harmful to periodontal condition and physical health but is not completely without risk. The components of ENDS vapor have cytotoxic, genotoxic, and carcinogenic properties, and its usage may be associated with a wide range of oral health sequelae. The chemicals in ENDS increase the susceptibility to tooth decay, increase the risk of periodontal disease, peri-implant, and oral mucosal lesions. Nicotine aerosols from ENDS can be a potential risk factor for oral cancer due to the presence of carcinogenic components. Compared to smoking traditional cigarettes, the harm associated with ENDS use may be underestimated due to the reduced ability to control vaping behavior, ease of ENDS access, fewer vaping area restrictions, and better taste. Currently, the available evidence suggests that ENDS may be a safer alternative to traditional tobacco products. Though most oral symptoms experienced by ENDS users are relatively mild and temporary compared to traditional cigarettes, the dangers of ENDS still exist. However, further research with longer follow-up periods is required to establish the long-term safety of ENDS.

## 1 Introduction

The World Health Organization has declared tobacco smoking to be the leading preventable cause of death worldwide (1). Tobacco contains more than 7,000 toxic chemicals, including several known human carcinogens, and causes more than 7 million deaths each year (2–4). Since the harm associated to cigarettes has been widely recognized by the public, the year 2006 saw the emergence of novel nicotine application devices that are similar in shape and function to conventional cigarettes and have been claimed to provide a more convenient and healthier alternative. These nicotine application devices are known as electronic cigarettes, vapes, e-hookahs, or electronic nicotine delivery systems (ENDS) (5, 6). In this review, for the sake of consistency, all cigarette-like devices that deliver nicotine are referred to as ENDS.

Patented in China in 2003 and introduced into Europe and North America in 2006, ENDS have become increasingly popular (7, 8). ENDS have long been marketed and advertised as being free of harmful ingredients such as tar and particulates, as well as being odorless, and have been promoted as a safe alternative for smokers who have difficulty quitting tobacco use (9, 10). ENDS are also marketed to the younger generation, and there are fewer restrictions on youth use of ENDS compared to traditional cigarettes (11). This view of ENDS as an acceptable alternative to tobacco smoking has now become widespread in society through advertising and peer recommendations. It was reported that the prevalence of current ENDS use among high school students was 27.5 percent, and middle school students was 10.5 percent (12). In 2019, among high school students, 4.11 million individuals admitted to using ENDS within the last month, whereas among middle school students, this number was 1.24 million, and for the first time, the total number of middle and high school students who reported using ENDS exceeded 5 million (12).

A growing number of countries are trying to regulate the sale of ENDS in the same way as tobacco products in an effort to reduce their use among young people (11, 13). The Indian government has approved an executive order banning the manufacture, import, export, sale, and advertising of ENDS (14). The US Department of Health and Human Services has also announced a ban on the sale of non-tobacco flavored ENDS products to curb the growing trend of vaping among teenagers (15). China also has a tobacco control campaign underway that aims to reduce the smoking rate among people aged 15 years and older to <24.5% by 2022 and 20% by 2030, and online sales of ENDS are banned in the same way as tobacco products (8, 16, 17).

In the field of stomatology, tobacco use is considered a risk factor for periodontal disease, implant-related disease, mucosal disease, and oral cancers (18–21). However, the consensus on the effects of ENDS on oral health needs to be further strengthened (22). Studies have shown that these devices still have harmful effects on the respiratory (23, 24), nervous (25, 26), reproductive (27, 28), and digestive systems (29), and they should be considered as carcinogenic factors due to their nicotine content and other additives (30–32). In spite of this, the risks of ENDS may also have been underestimated due to the vast amount of advertising claiming that ENDS pose less or no harm. This article aims to review the existing evidence about the effects of ENDS on oral health, to determine the differences between the effects of ENDS and of traditional cigarettes, to summarize the risks of ENDS for oral health, to provide a warning to the public against heavy use of these systems, and to provide ideas for future research.

## 2 Methodology

To ensure the objectivity, openness and reproducibility of this study, we have only included literature in English in our search, and gray literature which have not been published through traditional academic channels was not included. The PubMed and Cochrane Library electronic databases were searched for articles published between January 2012 and October 2022.

The search terms were the MeSH word “electronic nicotine delivery system.” When we need to explore the relationship between ENDS and specific domains, new search terms (i.e., caries, periodontal disease, tooth discoloration, dental prosthesis, peri-implantitis, oral mucosa, maxillofacial tumors, or maxillofacial injuries, etc.) were added to narrow the search scope when necessary, using the Boolean operator “AND,” to retrieve specific information about the relationship of ENDS with them.

Two authors independently screened the titles and abstracts of the identified studies and retrieved the full text of potentially eligible articles that met the inclusion criteria. To meet the analysis requirements and reduce deviation, studies were eligible if the following criteria were met: (1) Clinical studies related to ENDS, and its contents should include clinical or public health studies comparing ENDS users with regular tobacco users, and or with non-smokers. (2) The research content needs to be relevant to oral-related diseases. The exclusion criteria were: duplicate literature, pure animal or laboratory studies, unregistered clinical studies, literature with conflicts of interest, articles for which the full text was not available, incomplete data, and inability to obtain data from the original authors of the study (The literature search and screening process is illustrated by flow chart in Supplementary Figure 1).

## 3 Discussion

### 3.1 General composition of ENDS

ENDS are electronic devices that mimic cigarettes and offer an experience that is very similar to smoking. An ENDS is essentially a portable vaporizer shaped like a pen or a USB stick, powered by a rechargeable lithium polymer battery that heats nicotine oil and converts it into vapor, allowing the user to inhale the vapor in a way similar to smoking conventional cigarettes (Table 1).

**Table 1:** The general difference between cigarettes and ENDS.

	**Cigarettes**	**ENDS**
Ingredients	Tobacco	Nicotine; Solvent (Glycerin, Propanediol, Propylene glycol etc.)
Flavoring	Scarcely any	Diversified
Prevalence trends	Stable, slowly declining	Fast increasing
Content of inhalation	Smoke from burning tobacco	Aerosol from heated e-liquid
Product of combustion	Large amounts of particulate matter, carbon monoxide, tar	None
Carcinogens	More than 70 carcinogens in combustion	Greatly reduced with unknown long-term effects

Mainstream ENDS generally have pre-filled nicotine pods. It is likely that there are tens of thousands of e-liquid formulations available, all of which contain a variety of spices and flavors, unlike traditional cigarettes. The main ingredients in e-liquid formulations are nicotine, flavoring, and a matrix. The matrix consists mainly of glycerin, propanediol (propylene glycol), or a mixture. Propanediol is a colorless liquid with a slightly sweet taste. When a propanediol aerosol is formed by heating and atomization, decomposition products may be formed, including acetic acid, lactic acid, and propanol (33, 34). Propanediol has certain hygroscopic properties and combines readily with saliva, resulting in dry mouth. Glycerin is also a colorless liquid that is 60% sweeter than sucrose but is not metabolized by caries-causing bacteria. These ingredients in e-liquid formulations can demineralize enamel and increase susceptibility to caries.

There is great variation in the nicotine content of e-liquid pods (35). However, an e-liquid pod typically contains the same amount of nicotine as 2–3 packs of regular cigarettes. A typical pod can provide 200–400 puffs, and a typical user takes on average 100–150 puffs per day. Therefore, a moderate user will use one e-liquid pod every 2–3 days; however, a heavy user may use one pod per day (36). However, according to results from Yingst et al. (37), regardless of the generation of ENDS products, they delivered less nicotine compared to combustible cigarettes (38). We also note that these two studies were not from the same center, so more evidence of nicotine delivery comparison from between from ENDS and traditional combustible cigarettes may be needed.

### 3.2 Relative advantages of ENDS

ENDS are claimed to be less harmful to health, to be ash-free, to not require burning, to not generate ash or residues affecting the environment, and to not emit disagreeable odors. The smoke has no smell or a sweet smell, it evaporates quickly, and does not cause bad breath, making it more friendly to non-smokers. Unlike traditional cigarettes, ENDS do not contain tar, a chemical widely considered a carcinogen (39).

Some studies claimed that ENDS can help smokers to change their smoking habits. As an alternative to cigarettes, ENDS may relieve smokers from experiencing withdrawal symptoms and provide them with more choices (40). Smokers can switch to e-liquid products with different flavors and have a better quitting experience (41). Smokers switching from harmful tobacco smoking to (purportedly) less harmful ENDS have claimed to experience an overall health improvement and fewer respiratory infections. A study by Lee et al. (42) claimed that smoking may critically exacerbate COVID-19-related inflammation or increase susceptibility to COVID-19, while ENDS do not. Notley et al. (41) also indicated that the flavored e-liquids are an important aspect of young people's taste for ENDS and may encourage young people to switch from tobacco smoking to the less harmful ENDS. Moreover, a review from Holliday et al. (43) concluded that among smokers who use ENDS as a smoking cessation aid, the benefits of cessation may outweigh the negative effects of ENDS use on oral health, especially in the short term.

Though agencies such as the Food and Drug Administration (FDA) have not approved these vaping devices as aids for smoking cessation, FDA has approved the Nicotrol inhaler for this purpose (43). Nicotrol is not exactly an ENDS as it does not electronically vaporize the nicotine; nonetheless, it does deliver nicotine vapor. Rather, Nicotrol is classified as a nicotine replacement therapy (NRT).

In addition, the UK and Public Health England indicated that vaping poses a small fraction of the risks of smoking. A 2022 report (44) in the Cochrane Library states that “There is high-certainty evidence that Electronic cigarettes (ECs) with nicotine increase quit rates compared to NRT and moderate-certainty evidence that they increase quit rates compared to ECs without nicotine.”

### 3.3 Risk factors of ENDS products

The nebulizer of ENDS is generally composed of coil and wick materials, usually containing copper, silver, zinc, tin, nickel-chromium alloy, chrome-aluminum alloy, or other metal materials (44). Williams et al. (45) found that after repeated heating and cooling cycles, metal components would penetrate into the liquid, and these components had certain toxicity. Kankanamage et al. (46) claimed that tin, copper, nickel, other heavy metal elements, and silicate substances could be detected in ENDS aerosols, and the copper content was six times higher than that of cigarette smoke, which may aggravate the oxidation and damage to DNA. On the other hand, Palazzolo et al. (47), claimed that trace metals in ENDS aerosol were lower than in conventional mainstream cigarettes, and only nickel in ENDS aerosol was higher than in control cigarettes. The most likely source of nickel in this aerosol is the core component, especially the coils.

Although the effects of the flavoring in ENDS on human health have not been thoroughly studied, existing studies indicate that most flavoring can pose significant health risks if used for a long time, especially sweet flavoring (48). Substances that have been identified by the American Institute of Flavor and Extract Manufacturing to be contained in ENDS flavorings are potential respiratory irritants or poisons (49), and Khlystov et al. (50) also claimed that the flavoring agents in ENDS are an important factor in the production of toxic carbonyl and other substances.

### 3.4 Effects of ENDS on teeth

ENDS may have an adverse impact on teeth. Although they do not contain tar, which is a well-known carcinogen and stains the teeth, the interaction between tooth enamel and substances like glycerin, propanediol, and nicotine in e-liquid may also cause tooth decay and discoloration.

#### 3.4.1 Relationship with caries

The combination of glycerin and flavoring agent in e-liquid pods may be associated with a four-fold increase in microbial adhesion and a two-fold increase in biofilm formation (33). Sticky aerosols produced by heated e-liquid encourage the caries-causing bacterium Streptococcus mutans to adhere to tooth enamel, leading to demineralization and caries.

Rouabhia et al. (51) reported that ENDS cause increased growth and adherence of S. mutans. Moreover, they found an increase in the mass of biofilm from 8 ± 0.5 mg to 47 ± 5 mg after a six-round exposure to ENDS as well as increases in the expression of glucosyl transferase and glucan-binding genes. A cross-sectional study of National Health and Nutrition Examination Survey data for 2017–2018 by Vemulapalli et al. (52) found that combined use of ENDS and tobacco products was associated with an increased risk of caries. According to recent research, the aerosolized e-liquid emitted from a heated ENDS may have physicochemical properties similar to those of high-sucrose gelatinous confectionery and acidic drinks. The viscosity of e-liquids combined with the chemicals in the flavorings may therein increase the risk of dental caries (53).

#### 3.4.2 Teeth discoloration

Though ENDS manufacturers claim that the tooth discoloration caused by ENDS is significantly less pronounced than that caused by tar-containing tobacco products, studies reveal that the aerosol produced by ENDS may cause color changes in teeth and dental prosthesis or fillings. Researchers used spectrophotometry to compare enamel specimens after exposure to e-liquid aerosol according to flavor and nicotine content and found discoloration of enamel *in vitro*. The chemical substances used to flavor e-liquids, such as menthol, may be the reason behind the color alteration of enamel (54).

ENDS may also affect the color stability of tooth restoration materials. Vohra et al. (55) reported that exposure to ENDS produced discoloration levels on prosthodontic materials that was similar to that associated with the smoking of tobacco products. In their study, which included ceramics and resin materials with different surface properties, discoloration caused by both ENDS and conventional tobacco cigarettes was below clinically perceptible levels for ceramic materials but was visually perceptible for composite resins. However, other studies claimed that ENDS have an alleviating effect on tooth color compared with tobacco. Dalrymple et al. (56) reported that tobacco exposure significantly increased the level of discoloration in samples of bovine enamel, whereas exposure to ENDS products resulted in discoloration values comparable to those in non-exposed controls.

Considering the available evidence, no firm conclusions can be drawn about the effect of ENDS on tooth discoloration. Although the effect of ENDS on tooth color may be less pronounced than that of traditional cigarettes, long-term, high-dose exposure to ENDS does cause tooth discoloration. The rate of color change varies between natural tooth enamel and restoration materials composed of resin or ceramics. The rate of color change may also be affected by the brand and flavor of the e-liquid.

#### 3.4.3 Effects on periodontal tissue

There is a broad consensus that cigarette smoking has a considerable impact on oral periodontal health. Smokers have poor oral hygiene, and the tar in tobacco is conducive to pigmentation and adhesion of bacteria on the tooth surface, resulting in accumulation of plaque and increased calculus, gingivitis, and periodontitis. Nicotine can cause vasoconstriction and decreased blood flow, which results in a reduced oxygen and blood supply to the gums and a reduction in the ability of the gums to remain healthy.

Porphyromonas gingivalis (P. gingivalis) is often the therapeutic factor and initiating factor of periodontal diseases (57), such as gingivitis and periodontitis, which eventually lead to more severe systemic effects, such as diabetes, cardiovascular disease, and even Alzheimer's disease (58). According to the available research results, the counts of periodontopathogenic bacteria in the subgingival oral biofilm are similar in individuals who smoke combusting cigarettes, and those who use ENDS (59), and the effect of ENDS on P. gingivalis-induced periodontitis remains undetermined (60).

Tobacco affects immune status by decreasing immunoglobulin levels, including those of IgA, IgM, and IgG in serum, and IgA in saliva (61, 62). Periodontal parameters such as bleeding on probing, probing depth, and attachment loss are also affected. Although ENDS are advertised as being tar-free and less harmful to periodontal health than tobacco products, these claims are not necessarily borne out in reality. A study by Yang et al. (63) indicated that ENDS users exhibit an altered oral microbiome, and dual use of ENDS and conventional cigarettes is associated with the presence of several known pathogenic microbes. A cross-sectional study based on 8,129 participants indicated that ENDS ever users and current users had higher odds of self-reported periodontal disease (OR = 1.43, 95% CI: 1.18, 1.73) compared to non-users after adjusting for smoking and potential confounders (64).

Relevant studies indicated that patients using ENDS have better preservation of alveolar bone height compared with traditional smokers. However, whether there is any difference between ENDS users and non-smokers on alveolar bone height preservation is controversial. BinShabaib et al. (65) claimed that the marginal bone loss was significantly higher in smokers and ENDS users than in non-smokers. A study by ArRejaie et al. (66) claimed that the marginal bone loss level was significantly higher in tobacco smokers than in ENDS smokers or non-smokers. A study by Xu et al. (67) also indicated that bleeding on probing and probing depths similarly increased in ENDS smokers, tobacco smokers, and non-smokers, but clinical attachment loss uniquely increased in ENDS smokers. As increased levels of proinflammatory cytokines suggest a greater peri-implant inflammatory response, Mokeem et al. (68) found no significant difference in probing depth, clinical attachment loss, mesial or distal marginal bone loss, or salivary IL-1β and IL-6 levels between ENDS users and never-smokers.

As there is not enough evidence to fully characterize the impacts of vaping on periodontitis (60), further comprehensive evaluation of the long- and short-term effects of e-cigarettes on periodontal health is needed. Especially large-sample randomized prospective cohort studies, to reveal the mechanism associated with ENDS exposure on periodontal tissues and cells, immune system and microorganisms related to periodontal disease.

### 3.5 Effects on oral implants

At present, there are very few large-scale clinical studies on the effects of exposure to ENDS on oral implants, and the follow-up duration in the existing studies has been insufficient.

In a study of patients with implants by Ibraheem et al. (69), plaque index and probing depth values were significantly higher in cigarette smokers, waterpipe smokers, and ENDS users than in non-smokers. The Receptor Activator of Nuclear Factor-κB Ligand and osteoprotegerin levels in gingival crevicular fluid were also higher in users of any type of tobacco or alternatives than in non-smokers; however, there was no significant difference between smokers and ENDS users.

In contrast, Alazmi et al. (70) found no significant difference in the functional stability of dental implants between ENDS users and non-smokers. Moreover, in their study, there was no significant between-group difference in the plaque index, bleeding on probing, probing depth, or mesial or distal crestal bone levels around the implants after 8 years of follow-up. The authors suggest that if patients can adopt strict oral hygiene measures, dental implants can demonstrate stable clinical and radiographic status and remain as functionally stable in ENDS users as in non-smokers. A study by Sinha et al. (71) found that ENDS users had higher levels of inflammatory mediators, indicating a greater amount of localized inflammatory destruction of tissue and compromised peri-implant structures.

Overall, ENDS appear to be more implant-friendly than conventional tobacco cigarettes; however, whether or not there is a difference in the effect on implants between ENDS and non-smokers needs to be determined by further clinical evidence.

### 3.6 Effects on the oral mucosa

Many of the carcinogens present in tobacco are not present in the aerosol generated by ENDS, which makes ENDS appear to be less damaging to the oral mucosa. However, it should be noted that fogged nicotine can cause symptoms, such as dry mouth and throat, and can also burn the oral mucosa (72, 73).

Chaffee et al. (74) assessed the relationship between the prevalence of xerostomia and the use of ENDS, marijuana, and combustible tobacco in a cohort of adolescents recruited from public high schools in Northern California. Symptoms of dry mouth were more prevalent in cannabis users (19%) and tobacco users (19%) than in frequent ENDS users (14%) or non-smokers (14%), indicating that oral symptoms are less likely in ENDS users than in traditional smokers.

Other studies indicate that other oral mucosal symptoms, including black tongue, burns, nicotine stomatitis, and hairy tongue are significantly more common in ENDS users than in ex-smokers. The effects of ENDS on the oral mucosa appear to be milder and more transient than those associated with tobacco smoking, and their specific effects are related to the flavors used. Menthol and cinnamon appear to cause more severe oral irritation while citrus, sour, cola-like, and custard flavors are associated with more severe throat symptoms (75). Nicotine increases short-term blood flow to the mucosal tissues, suggesting that menthol may mask respiratory irritation caused by high nicotine levels.

Studies have also shown that ENDS users experience fewer adverse reactions, and former smokers report improvements in taste and bad breath (76). Non-smokers who use ENDS report higher levels of oral discomfort than those who use nicotine substitute therapy. However, ENDS can also cause xerostomia, burning, and other symptoms. Nonetheless, due to the reduction in associated irritants, carcinogens, and hot airflow, in general, the oral mucosa conditions are considerably improved in patients who switch from tobacco smoking to ENDS based on the evidence available (77, 78).

### 3.7 Potential effects on maxillofacial tumors

The World Health Organization has identified heated propylene glycol as a carcinogen, and the saliva of ENDS users has been found to contain carcinogens commonly associated with traditional smoking, including N'-nitrosonornicotine and thiocyanates (79, 80). Therefore, ENDS vapors could still have cytotoxic, genotoxic, and inflammatory effects. Many adverse effects could occur at the cellular level after long-term exposure to these harmful aerosols, including reduced cell proliferation and activity, changes in cell morphology and activity, cell apoptosis and necrosis, DNA damage, and increased transcription of pro-inflammatory cytokines, with an increased risk of carcinogenesis (30, 31, 81).

Cigarette smoke is a multifaceted chemical concoction encompassing several tobacco-specific nitrosamines (TSNA) (82). A plethora of evidence (83–85) suggests that TSNA is a significant pathogenic factor for lung, pancreatic, esophageal, and oral cancers in tobacco-using individuals. The most carcinogenic TSNA variants are nicotine-derived nitrosamine ketone and N-nitrosonornicotine (86). It has been reported that the detection of TSNAs in ENDS in the form of E-liquids and aerosols and elevated temperature accelerated a process whereby the levels of TSNA in e-liquids increased over time, particularly in the presence of nitrite and nicotine (87). Meanwhile, Farsalinos et al. (88) showed that the TSNA content in ENDS aerosol was correlated with the content in E-liquid. Despite this, TSNA levels are lower than those in combusting tobacco cigarettes (89).

The aerosols delivered by ENDS, which range in temperature from 130 to 350°C depending on the device, may also play a role in the carcinogenic potential of vaping (90). Apart from that, high temperatures can lead to the release of aldehydes and cause nicotine stomatitis, which is considered to be a precancerous lesion. Acrolein, a byproduct in ENDS, can induce oxidative stress, inflammation, and damage the endothelial cell barrier. Genetic disorders associated with carcinogenic pathways have also been found in oral tissue exposed to ENDS. Due to the lack of long-term prospective and large case-control studies, and the fact that ENDS users tend to present other established risk factors such as traditional smoking and alcohol consumption, it is difficult to assess the relative importance of ENDS as an independent risk factor for oral cancer.

### 3.8 Limitations of this review

Many ENDS users also smoke tobacco cigarettes, or have a long history of smoking. Therefore, the crossover between ENDS and conventional cigarette use makes isolating the effect of ENDS on oral health difficult. In addition, although we assessed the quality of all the included studies, we could not actually evaluate their authenticity or validity. More high-quality prospective studies are needed to discover evidence-based information to evaluate the long-term safety of ENDS with more depth.

## 4 Conclusions

Existing studies have shown that ENDS, while tar-free and containing less particulates than traditional cigarettes, still pose some risk for oral health. ENDS may increase susceptibility to caries, periodontal disease, and mucosal disease; and discolor teeth and prostheses. In addition, heated nicotine aerosols from ENDS can encompass several tobacco-specific nitrosamines that are considered a potential risk factor for cancer and induce detrimental changes in cells from the oral cavity.

Currently, existing evidence already supports the conclusion that electronic cigarettes are less harmful than traditional cigarettes. However, there is still little evidence on the short- or long-term effects of ENDS on oral health. The preliminary clinical studies available to date have not reported long-term effects or are still restricted to a small number of participants, which limits their validity. More research is needed to elucidate the impact of ENDS use on oral health.

## Author contributions

QZ: validation, investigation, and writing-original draft preparation. CW: conceptualization, resources, writing-review and editing, supervision, project administration, and funding acquisition. All authors reviewed and approved the final version.
