# Supplementary material for: The risk profile of electronic nicotine delivery systems, compared to traditional cigarettes, on oral disease: a review
Source: Front Public Health. 2023 May 15;11:1146949. doi: 10.3389/fpubh.2023.1146949 (PMC10226679; doi:10.3389/fpubh.2023.1146949)
Supplement: Supplementary file 1 [file Table_1.DOCX]

Supplementary 1： The flow chart of literature collecting and screening on ENDS

Specific Oral Domains：caries, periodontal disease, tooth discoloration, dental prosthesis, peri-implantitis, oral mucosa, maxillofacial tumors, or maxillofacial injuries, etc.

Full-text articles assessment

for inclusion

Records screening

“ENDS” + “Specific Oral Domains”

Cochrane Library

MeSH Word：“ENDS”

PubMed
